# Dealing with Chronic Non-Bacterial Osteomyelitis: a practical approach

**DOI:** 10.1186/s12969-017-0216-7

**Published:** 2017-12-29

**Authors:** Andrea Taddio, Giovanna Ferrara, Antonella Insalaco, Manuela Pardeo, Massimo Gregori, Martina Finetti, Serena Pastore, Alberto Tommasini, Alessandro Ventura, Marco Gattorno

**Affiliations:** 1 Institute for Maternal and Child Health, IRCCS “Burlo Garofolo”, Trieste, Italy; 2 University of Trieste, Via dell’Istria 65/1, 34100 Trieste, Italy; 3 Division of Rheumatology, Department of Paediatric Medicine, Bambino Gesù Children’s Hospital, IRCCS, Piazza di Sant’Onofrio, 4, 00165 Rome, Italy; 4 Pediatria 2, Istituto Gaslini, Via Gerolamo Gaslini, 5, 16148 Genoa, Italy

**Keywords:** Chronic Non-Bacterial Osteomyelitis, Chronic recurrent multifocal Osteomyelitis, Autoinflammatory syndrome, Magnetic resonance, Treatment, Bisphosphonate, Anti-TNFα treatment

## Abstract

**Background:**

Chronic Non-Bacterial Osteomyelitis (CNO) is an inflammatory disorder that primarily affects children. Although underestimated, its incidence is rare. For these reasons, no diagnostic and no therapeutic guidelines exist. The manuscript wants to give some suggestions on how to deal with these patients in the every-day clinical practice.

**Main body:**

CNO is characterized by insidious onset of bone pain with local swelling. Systemic symptoms such as fever, skin involvement and arthritis may be sometimes present. Radiological findings are suggestive for osteomyelitis, in particular if multiple sites are involved. CNO predominantly affects metaphyses of long bones, but clavicle and mandible, even if rare localizations of the disease, are very consistent with CNO diagnosis. CNO pathogenesis is still unknown, but recent findings highlighted the crucial role of cytokines such as IL-1β and IL-10 in disease pathogenesis. Moreover, the presence of non-bacterial osteomyelitis among autoinflammatory syndromes suggests that CNO could be considered an autoinflammatory disease itself. Differential diagnosis includes infections, malignancies, benign bone tumors, metabolic disorders and other autoinflammatory disorders. Radiologic findings, either with Magnetic Resonance or with Computer Scan, may be very suggestive. For this reason in patients in good clinical conditions, with multifocal localization and very consistent radiological findings bone biopsy could be avoided. Non-Steroidal Anti-Inflammatory Drugs are the first-choice treatment. Corticosteroids, methotrexate, bisphosphonates, TNFα-inhibitors and IL-1 blockers have also been used with some benefit; but the choice of the second line treatment depends on bone lesions localizations, presence of systemic features and patients’ clinical conditions.

**Conclusion:**

CNO may be difficult to identify and no consensus exist on diagnosis and treatment. Multifocal bone lesions with characteristic radiological findings are very suggestive of CNO. No data exist on best treatment option after Non-Steroidal Anti-Inflammatory Drugs failure.

## Background

Chronic Non-Bacterial Osteomyelitis (CNO) is a rare inflammatory disorder not related to infectious disease [1]. It was first described in 1972 by Giedion et al. [2] as a symmetric multifocal bone lesions; later, in 1980, Bjorksten B et al. [3] first used the term CNO in order to identify a clinical condition which is characterized by recurring episodes or persisting presence of chronic sterile osteomyelitis [4–6]. Multiple names have been used in literature to describe this disorder; these include chronic recurrent multifocal osteomyelitis (CRMO) in cases with extended multifocal involvement (often symmetric) and synovitis, acne, pustulosis, hyperostosis, and osteitis syndrome (SAPHO), which usually manifests in adolescent and adult patients and which distinguishes for skin involvement [7]. The terms CRMO and CNO are often used interchangeably. Although CNO is still considered a rare disorder, its incidence is probably underestimated. In fact, in a single center retrospective study, it has been recently demonstrated that the incidence of CNO is similar to infectious osteomyelitis [8]. For these reasons, in absence of standardized diagnostic work out and treatment guidelines, it is important to provide some clinical practical suggestion about the every-day clinical management of CNO patients.

## Main Text

### Pathogenesis

Since this is not the main aim of the manuscript, CNO pathogenesis will be only briefly discussed. Although CNO pathogenesis is still not clear, the hypothesis that the disorder could be sustained by infections was not confirmed by extensive microbiological analyses and uselessness of antibiotic treatment [4]. Findings indicate that pro-inflammatory cytokines such as IL-6 and TNF-a as well as anti-inflammatory (IL-10) [4, 9], IL-1β [10] and IL-10 [11] may play an important role in disease pathogenesis of CNO. In the last years, the hypothesis that CNO might be a genetic disease in the spectrum of autoinflammatory disorders has acquired even more importance. The strongest evidence comes from the so called syndromic forms of CNO: Majeed syndrome [12, 13], Cherubism [14], Hypophosphatasia [15] and Primary Hypertrophic Osteoarthropathy [16]. In addition, chronic osteomyelitis is a typical feature of two monogenic diseases caused by mutations of genes involved in the activation of the NLRP3 inflammasome or in the homeostasis of IL-1, namely pyogenic arthritis, pyoderma gangrenosum and acne (PAPA) syndrome [17] and the deficiency of IL-1 receptor antagonist (DIRA), [18] respectively. There is also some evidence for a genetic basis in non-syndromic or sporadic CNO [19]. Moreover, in largest cohorts of CNO patients, the prevalence of the disease among patients’ relatives was higher [20] and some reports have described families with multiple affected members [21] or have reported a high incidence of psoriasis, inflammatory bowel disease, and other chronic inflammatory conditions in first-degree family members of individuals with CNO suggesting that there is a significant genetic component to disease susceptibility [3, 6]. In mice, homozygous mutation of *PSTIPI2* gene results in an autoinflammatory disease very similar to CNO [22, 23].

### Clinical features

The clinical manifestations of CNO are highly variable. CNO typically presents with bone pain that is worse at night and occurs in the presence or absence of fever [20, 24]. The onset is typically insidious, and most children appear well.

Swelling and heat of the involved bone are not necessarily always present. In 30% of cases CNO involves the adjacent joint with the presence of exudate, synovial thickening and/or damage to the articular cartilage. The lesions may affect any bone segment. One to 20 sites can be affected at one time. The main sites of involvement in order of decreasing frequency are the lower extremities, pelvis, clavicle and spine [6, 20, 24]. Metaphyseal area is the most common bone site localization as well as the involvement of clavicle, mandible and sternum which is particularly suggestive of CNO [20]. The skull involvement has been described in the occipital bone in only one case. In this patient, however, the lesion was not present at time of diagnosis, but it developed after 1 year from diagnosis [25]. Skull involvement should always be considered a potential malignancy; in this case bone biopsy is mandatory.

Systemic symptoms are subtle and may be present in the form of low-grade fever, malaise, or poor growth. In this case, malignancies, most of all acute lymphoblastic leukemia, and inflammatory bowel disease must be ruled out. Current estimates suggest that approximately 25% of individuals with CNO have manifestations involving organ/systems other than bone [20]. The extra - articular manifestations include the skin (especially Psoriasis, Palmoplantar Pustulosis, Acne, Pyoderma Gangrenosum and Sweet Syndrome) and the bowel (Crohn Disease, Ulcerative Colitis, Celiac Disease) [26]. Renal involvement has been demonstrated in almost 10% of patients [27].

The disease may follow a chronic or recurrent disease course, often the course is prolonged over several years with periodic exacerbations [1–6]. The prognosis is generally good and provides self-resolution in a time ranging from months to years. However, recently complications of entity variable from mild to incapacitating have been described in a considerable percentage of cases (30 to 50%). In particular asymmetries of limb length, kyphosis, chronic spondylo-arthropathy, vertebral collapse and stunting for early closure of the growth-cartilages have been reported [6, 7, 24]. Monophasic disease is usually less severe and prognosis is excellent being, in most cases, almost a cosmetic problem.

### Diagnosis

CNO is a diagnosis of exclusion. Differential diagnoses include infections (septic osteomyelitis, typical and atypical mycobacterial infections, etc.), malignancies (primary bone tumors and leukemia/lymphoma), benign bone tumors (osteoid osteoma), trauma, metabolic disorders (including hypophosphatasia), other autoinflammatory disorders (DIRA, PAPA, Cherubism, etc.), osteonecrosis and osteopetrosis.

The most common clinical challenge is with acute bacterial osteomyelitis; in this case, however, pain and fever are usually present and, except for some rare circumstances, such as severe immunodeficiencies, the disease is always monofocal. In early stage of the disease, and in the monofocal course, the radiological assays may be undistinguishable and a trial with antibiotics is indicated. If there will be no response to antibiotic treatment, once ruled out infective complication (e.g. bone abscess), CNO should be taken into account. Malignancies should be considered in any patients with poor clinical conditions, with systemic features, with skull involvement or with suggestive radiologic lesions. Osteoma Osteoid has a very typical radiological pattern (nidus surrounded by dense bone) and nocturnal pain is almost even present. Hypophosphatasia is an inherited disorder that affects the development of bones and teeth. This condition disrupts bone mineralization, causing skeletal abnormalities similar to rickets. However, the forms of hypophosphatasia that appear in childhood or adulthood are typically less severe than those that appear in infancy and may present as genu varum or genu valgum, enlarged wrist and ankle joints, and an abnormal skull shape leading to CNO diagnosis. CNO may be present in other autoinflammatory disorders; however, in this case, many other clinical features may prevail such as arthritis, pustulosis, hepatomegaly, interstitial pneumonia, splenomegaly, fever and etc. Differential diagnosis are summarized in Table 1.

**Table 1:** Clinical characteristics of CNO compared with other bone diseases

	CNO	Bacterial Osteomyelitis	Malignancy	Osteoid Osteoma	DIRA	PAPA	Cherubism	Osteopetrosis
Multifocal Involvement	+++	–	+	–	+	−/+	+	+++
Pain	++++	++++	++++	++++	++	+++	++	++
Fever	+	+++	−/+	–	−/+	−/+	–	–
Skin Involvement	+	–	–	–	++++	+++	–	–
Articular Involvement	+	++	–	–	++++	++++	–	–
Bone Swelling	+++	−/+	++	–	++	++	++++	++
Renal Involvement	+	–	−/+	–	–	+	–	–
Hepatosplenomegaly	–	–	+	–	++++	–	–	++
Early Age of onset	−/+	+	−/+	–	++++	–	–	++
ESR/CRP elevation	+	+++	−/+	–	++++	++++	–	–
Leukocytosis	−/+	+++	−/+	–	++++	++++	–	–

Laboratory investigations may reveal mild elevation in white blood cell count and in inflammatory parameters (C-Reactive Protein; Erythrocyte Sedimentation Rate), but often these abnormalities are absent in CNO patients [20, 24]. Cultures of blood and bone are invariably negative, and sophisticated assays to identify evidence of a microbial etiology have been unsuccessful. Autoantibodies (antinuclear antibodies, rheumatoid- factor), as well as carriage of the HLAB27 allele, have the same prevalence in CNO patients when compared to healthy individuals. At present, no specific biomarkers are available for the diagnosis or prediction of flares in CNO patients. In 2007 Jansson et al. [24] proposed diagnostic criteria for CNO according to which diagnosis could be formulated if present 2 majors and one minor criteria or one major and three minors criteria. However, these criteria are not still internationally validated and accepted so far. A crucial role in the diagnosis of this condition is provided by imaging and biopsy.

### The role of radiology

Standard radiography of bones could not reveal characteristic changes in early CNO, while the presence of osteolytic lesions with a sclerotic edge in X-ray imaging is the key feature later. Clavicular lesions and mandibular often have a more prominent sclerotic appearance [28].

**Fig. 1:**
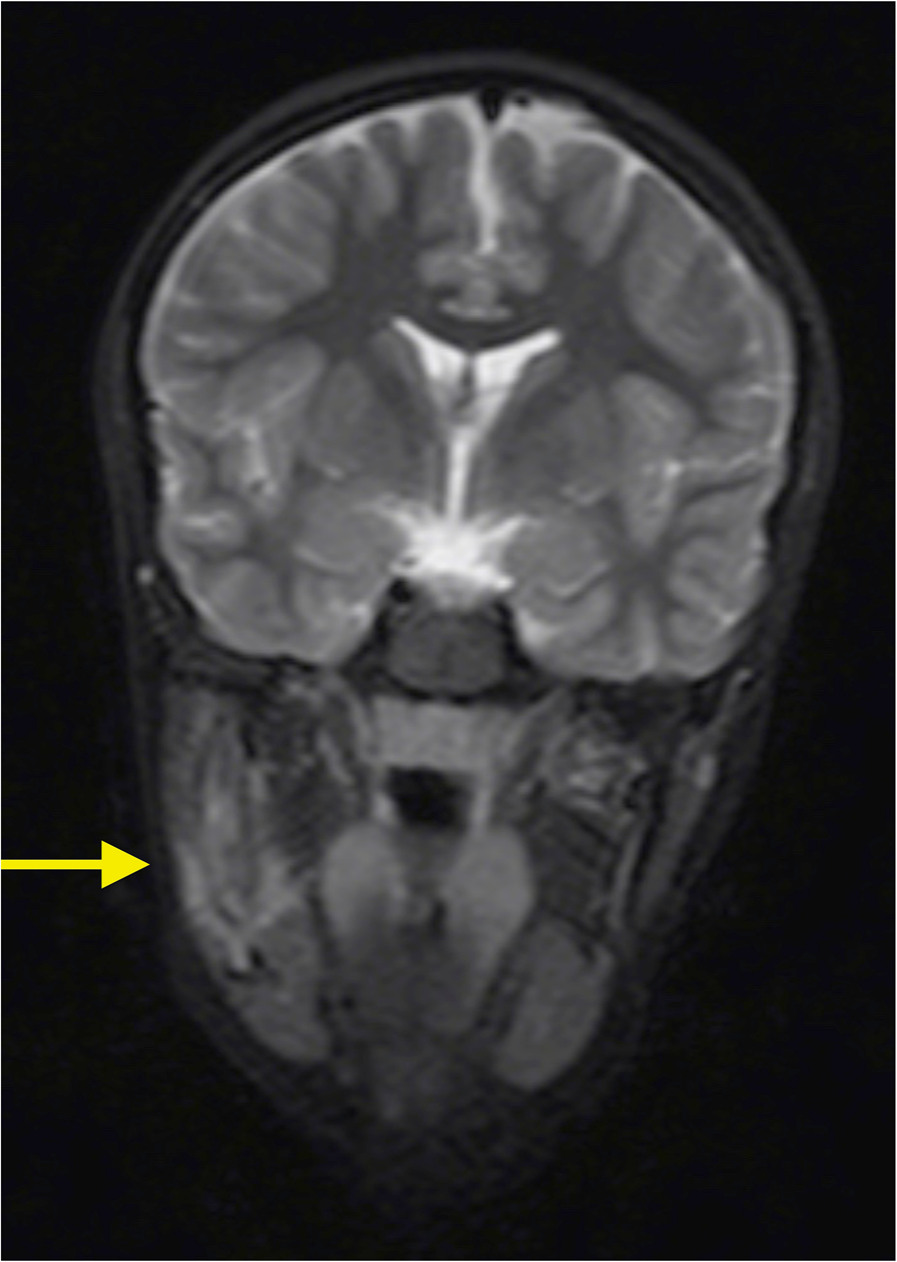
MR of mandible. Mandible edema and mandibular nerve canal enlargement (arrow) in a CNO patient

Cortical bone is usually unaffected and thickened but there are also reports of cortical defect mimicking tumor. The involvement of the mandible is often associated with mandibular nerve canal enlargement (Fig. 1). CNO is a systemic disorder that can affect multiple skeletal sites. Isotopic bone scan and/or whole-body Magnetic Resonance (MR) are the cornerstone for confirming the multifocal pattern of CNO, even if bone scan may be falsely negative in some cases. However, if isotopic bone scan may just confirm the presence of one or more foci of inflammation, MR may also add more information concerning the types of lesions being the more sensitive and accurate radiological examination at CNO diagnosis.

The typical MR findings are the presence of bone cortical thickening, lytic lesions with sclerosis and bone edema. Moreover, MR is particularly important in the early stages of the disease for its ability to detect bone edema and also asymptomatic bone lesions [29], before osteolysis and/or sclerosis can be detected. On the other hand, it should be mentioned that, due to its high sensitivity, this technique might lead to an over-interpretation of some bone lesions that, especially in pediatric age, can be related to normal bode growth or accidental traumatic events. This issue should be taken into careful consideration if the site of the bone biopsy is chosen on the basis of the MR images. Due to the lack of ionizing radiation, total body MR (with STIR sequences) is currently used to monitor the evolution of the bone lesions during the follow-up. Again, due to its high sensitivity MR might provide signs of possible bone activity in a subgroup of patients which did not complain any clinical manifestation or bone pain and could be considered in clinical remission [30]. For this reason it is not clear if the evidence of radiological disease activity in spite of a persistent clinical and laboratoristic, remission should be taken into consideration for patient’s treatment strategy or, vice versa, if it could lead to an over-treatment in some patients. For this reason, longitudinal MR control could be useful in particular among patients with a more severe disease course and resistant to ongoing treatments, and in case of the involvement of some specific sites, such as the mandible or the spine, which is traditionally characterized by a higher rate of complication such as scoliosis or kyphosis.

### The role of biopsy

Although no formal guidelines are so far available, a biopsy of the bone lesion is usually performed, mainly to exclude other causes. In CNO bone biopsy shows signs of inflammation in the absence of infection. The composition of cellular infiltrates at the sites of inflammation is strictly correlated to the “age” of biopsied lesions. Neutrophils are predominant in early lesions, whereas lymphocytes, macrophages and plasma cells can be detected during the later course of the inflammatory. The final stage of the lesion is characterized by the predominance of fibrosis. The cultures of the biopsy are always negative [4].

Although histological findings are specific, the main role of the biopsy is to rule out malignancy such as histiocytosis, Ewing sarcoma, osteosarcoma, leukemia and lymphoma. All these disorders should be considered in the differential diagnosis of persistent bone pain in all age groups.

**Fig. 2:**
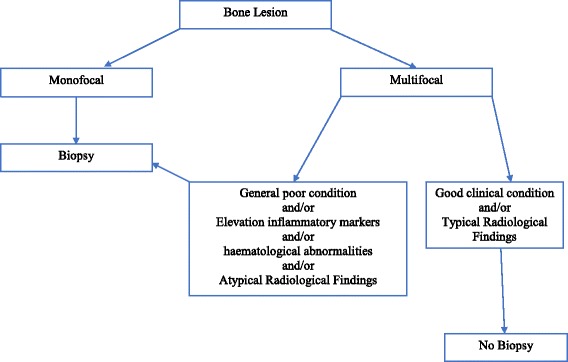
Suggested diagram to perform or not perform bone biopsy in a patient with suspected CRMO

Recently, Jansson et al. proposed a clinical score that could facilitate the diagnosis and treatment process, especially with respect to the decision on whether to carry out invasive procedures required for diagnosis of these diseases [31]. Even if the Jansson Score is not still widely validated, it is suggested that patients with a score > 39 could not undergo biopsy. Our practical approach is to perform a biopsy in all patients with poor general conditions, persistent and significant elevation of acute phase reactants and/or hematological abnormalities (anemia, alteration in leukocyte or platelet counts), and all those patients with unifocal or atypical (i.e. skull) bone involvement. In these cases, the biopsy may be performed in the most accessible lesion. On the contrary, the decision to perform the biopsy can be postponed in those patients with good general conditions, with slight elevation of acute phase reactants, involvement of multiple and/or typical and bone sites, typical radiological findings and favorable response to Non-Steroidal Anti-Inflammatory Drugs (NSAIDs) treatment (Fig. 2). In any case, further studies are needed to clarify the role of biopsy; to now the decision to perform the biopsy remains a physician related decision based on his expertness and knowledge toward CNO diagnosis.

### Treatment

Generally accepted treatment protocols for CNO do not exist and the treatment of CNO has been largely empiric. A number of retrospective assessments of response to treatment in case reports or small series are available in the literature. Neither Guidelines nor expert consensus treatment do exist for CNO so far; however our group has recently proposed a suggested treatment protocol base exclusively on their clinical expertise [32].

The first line treatment is usually NSAIDs, which have been demonstrated useful for pain control and inducing remission in a percentage of patients varying from 43 to 83% [24, 33]. The NSAIDs more frequently used are naproxen, indomethacin and, especially, in those patients with concomitant inflammatory bowel disease, sulfasalazine. The prospective use of NSAIDs has been evaluated in a single study performed in thirty-seven CNO patients. A favourable clinical course identified as symptoms free status was reported in 43% of patients taking naproxen at 1 year of follow-up; moreover, the total number of clinical detectable lesions was significantly reduced. Mean disease activity estimated by the patient/physician and the physical aspect of health-related quality of life including functional ability (global assessment/childhood health assessment questionnaire and childhood health assessment questionnaire) and pain improved significantly [33]. At least 1 month trial is needed in order to determine their failure. However, it is important to underline that NSAIDs did not seem to be sufficient for vertebral involvement or in case of peripheral arthritis. In case of NSAIDs failure, a single course of corticosteroids could be beneficial at the beginning of the disease; some others suggest their use only in those cases unresponsiveness to NSAIDs, in relapsing diseases or in severe clinical involvement [33, 34]. In this case, a bone biopsy should precede the use of steroid; prednisolone is usually the drug of choice.

TNF-alfa inhibitors have also been used in CNO patients. Infliximab was the first biologic treatment used [35] in an 18-years old with relapsing CNO. Etanercept has also been demonstrated effective in a patient with active disease despite previous treatment options, together with Methotrexate (MTX) [36]. Some large cohorts of patients report their use in a small sample of patients, usually <10%, who did not achieve clinical remission with previous treatment. In these patients, their use was usually of benefit [20, 24].

In the last years, the use of bisphosphonate was found to be effective and safe in the treatment of CNO. Most part of data about bisphosphonate use is based on case reports or small case series [37]. The most common used molecule is pamidronate, but alendronate has been reported to be safe and useful as well [38]. Bisphosphonates have demonstrated to be effective not only in controlling pain, [39] but recently it has also been demonstrated their efficacy in the resolution of bone lesions assessed by whole body magnetic resonance imaging [40]. At the moment it is not clear whether treatment option is better. Wipff et al. suggested that CNO patients may be divided into three categories of clinical severity. The patients with a mild phenotype presented a high remission rate irrespectively from treatment and use of pamidronate or TNF inhibitors; on the contrary, among the severe phenotype, the rate of clinical remission was lower despite high percentage of patients undergoing treatment with biphosphonate or TNF inhibitors [20]. In our experience bisphosphonate were the most useful treatment option irrespectively of previous treatment or clinical features [27]; in fact we have demonstrated that bisphosphonates may lead to remission the 73% of CNO patients after NSAIDs and steroids failure. For this reason, we would suggest that bisphosphonate could be considered the first treatment options for CNO patients when NSAIDs failed, especially when the spine is involved. Their safety is debated. Although to date, jaw osteonecrosis has never been reported in pediatric patients with CNO, periodic oral controls are recommended. In patients with concomitant gastro-intestinal or articular involvement (synovitis, spondylitis), sulfasalazine, MTX or anti-TNF treatment can be considered as treatments of choice. Data reporting clinical studies on CNO treatment are summarized in Table 2.

**Table 2:** List of manuscripts reporting data about response to treatment of patients with CNO

Reference	Nr. patients	Treatment	Response to treatment
Wipff J et al., 2015 [20]	178	NSAIDs	126/178 (71%): clinical response
		Sulfasalazine	7/17 (41%): clinical response
		Methotrexate	3/8 (37%): clinical response
		Bisphosphonates	6/8 (75%): clinical response
		Anti-TNFα	8/9 (89%): clinical response
Jansson A et al., 2007 [24]	89	NSAIDs	64/77 (83%): clinical response
		Steroids	13/13 (100%): transient response
		DMARDs	6/6 (100%): no response
		PAM	1/4 (25%): clinical response
			1/4 (25%:) partial response
			2/4 (50%): no response
Kaiser D et al., 2015 [45]	41	NSAIDs	21/37 (57%): clinical response
		Methotrexate	6/7 (86%): no response
		Bisphosphonates	1/5 (20%): clinical response
			1/5 (20%): partial response
		Etanercept	2/8 (25%): clinical response
Beck C et al., 2010 [33]	37	Naproxene	16/37 (43%) clinical response
		Indomethacin	4/7 (57%) clinical response
		Diclofenac	9/12 (75%) clinical response
		Others NSAIDs	6/19 (32%) clinical response
		Sulfasalazine	4/5 (80%) clinical response
		Steroids	4/4 (100%) clinical response but recurrence during dosage tapering
Roderick M et al., 2014 [40]	11	PAM	8/11 (73%) clinical response
Miettunen PM et al., 2009 [46]	9	PAM	9/9 (100%) clinical and radiological response
Gleeson H et al., 2008 [39]	7	PAM	6/7 (86%) clinical response
Hospach T et al., 2010 [47]	7	PAM	7/7 (100%) clinical response and radiological improvement
Kerrison C et al., 2004 [48]	7^a^	PAM	7/7 (100%) clinical remission
Batu ED, et al., 2015 [49]	5	Etanercept	5/5 (100%) clinical response
Simm PJ et al., 2008 [37]	5	PAM	4/5 (80%) clinical response and radiological improvement
Eleftheriou D et al., 2010 [50]	4	anti-TNFα	2/3 clinical response to infliximab, 1/3 response to adalimumab

*DMARDS* Disease-modifying anti-rheumatic drugs (Methotrexate or Azathioprine), *NSAIDs* Non-steroidal anti-inflammatory drugs, *PAM* Pamidonate

^a^patients with SAPHO

All monogenic forms of CNO (DIRA, Majeed syndrome and PAPA) respond really well to anti-IL1 blockade, [41–43] and, even if its role it is not clear in CNO, it has been recently demonstrated that Anakinra may be a possible therapeutic alternative in patients with refractory CNO [44].

The lack of longitudinal, placebo-control large studies on the different possible therapeutic strategies unable us to indicate an evidence-based approach to the treatment of this enigmatic and protean condition. Thus, the optimal treatment strategy for CNO remains to be determined.

## Conclusions

CNO is a rare disorder and many considerations about diagnosis and treatment remain to be clarified. However, clinical features, the presence of multifocal lesions and radiologic features may help in diagnosis avoiding bone biopsy. NSAIDs remain the first treatment option, while bisphosphonates and TNF-alpha inhibitors could be considered the best second line treatment option. Even if CNO is often a benign disease, it can lead to severe and persistent complications.

## Abbreviations

CNO: Chronic non-bacterial osteomyelitis
CRMO: Chronic recurrent multifocal osteomyelitis
DIRA: Deficiency of IL-1 receptor antagonist
MR: Magnetic resonance
NSAIDS: Non-steroidal anti-inflammatory drugs
PAPA: Pyogenic arthritis, Pyoderma gangrenosum and acne syndrome
SAPHO: Synovitis, Acne, Pustulosis, Hyperostosis, and Osteitis syndrome
